# Prognostic significance of non-infarcted myocardium correlated with microvascular impairment evaluated dynamically by native T1 mapping

**DOI:** 10.1186/s13244-022-01360-y

**Published:** 2023-03-20

**Authors:** Bing-Hua Chen, Dong-Aolei An, Chong-Wen Wu, Ting Yue, Matthew Bautista, Erika Ouchi, Jian-Rong Xu, Jiani Hu, Yan Zhou, Jun Pu, Lian-Ming Wu

**Affiliations:** 1 Department of Radiology, Renji Hospital, School of Medicine, Shanghai Jiao Tong University, No.160 PuJian Road, Shanghai, 200127 P. R. China; 2 Department of Radiology, Wayne State University, Detroit, MI 48201 USA; 3 Department of Cardiology, Renji Hospital, School of Medicine, Shanghai Jiao Tong University, No.160 PuJian Road, Shanghai, 200127 P. R. China

**Keywords:** Myocardial infarction, Magnetic resonance imaging, Ventricular remodeling, Fibrosis, T1 mapping

## Abstract

**Objectives:**

This study aimed to investigate the influence of microvascular impairment on myocardial characteristic alterations in remote myocardium at multiple time points, and its prognostic significance after acute ST-segment elevation myocardial infarction (STEMI).

**Methods:**

Patients were enrolled prospectively and performed CMR at baseline, 30 days, and 6 months. The primary endpoint was major adverse cardiac events (MACE): death, myocardial reinfarction, malignant arrhythmia, and hospitalization for heart failure. Cox proportional hazards regression modeling was analyzed to estimate the correlation between T1 mapping of remote myocardium and MACE in patients with and without microvascular obstruction (MVO).

**Results:**

A total of 135 patients (mean age 60.72 years; 12.70% female, median follow-up 510 days) were included, of whom 86 (63.70%) had MVO and 26 (19.26%) with MACE occurred in patients. Native T1 values of remote myocardium changed dynamically. At 1 week and 30 days, T1 values of remote myocardium in the group with MVO were higher than those without MVO (*p* = 0.030 and *p* = 0.001, respectively). In multivariable cox regression analysis of 135 patients, native_1w_ T1 (HR 1.03, 95%CI 1.01–1.04, *p* = 0.002), native_30D_ T1 (HR 1.05, 95%CI 1.03–1.07, *p* < 0.001) and LGE (HR 1.10, 95%CI 1.05–1.15, *p* < 0.001) were joint independent predictors of MACE. In multivariable cox regression analysis of 86 patients with MVO, native_30D_ T1 (HR 1.05, 95%CI 1.04–1.07, *p* < 0.001) and LGE (HR 1.10, 95%CI 1.05–1.15, *p* < 0.001) were joint independent predictors of MACE.

**Conclusions:**

The evolution of native T1 in remote myocardium was associated with the extent of microvascular impairment after reperfusion injury. In patients with MVO, native_30D_ T1 and LGE were joint independent predictors of MACE.

**Supplementary Information:**

The online version contains supplementary material available at 10.1186/s13244-022-01360-y.

## Key points

- Microvascular impairment correlated with native T1 of remote myocardium.
- Native T1 values of remote myocardium changed dynamically.
- Native_30D_ T1 and LGE were joint independent predictors of MACE.

**Supplementary Information:**

The online version contains supplementary material available at 10.1186/s13244-022-01360-y.

## Introduction

In the course of the past 30 years, the mortality rate during the acute phase of ST-elevation myocardial infarction (STEMI) has declined steadily and appeared now to have reached a plateau at lower values when compared with those in the pre-reperfusion era [1]. However, the success of emergency coronary reperfusion therapy in STEMI is usually limited by tissue perfusion failure [2], which has been shown to increase the risk of future cardiovascular events [3]. Several published trials have provided evidence that MVO is the best predictor for prognostic value among all CMR parameters, including clinical scores, left ventricular ejection fraction, and infarct size [4–6]. Microvascular injury after acute myocardial infarction affects local T1 value [7]. However, in patients with acute myocardial infarction (AMI), myocardial tissue injury and cardiac remodeling extend beyond the region supplied by the culprit artery; they also affect the remote, non-infarcted myocardium [8].

Several studies have reported remote myocardium alterations in patients with AMI, both in animal and clinical studies [9–12]. A study by Carrick et al. [13] showed that in remote zone native T1 mapping, early changes may be associated with the occurrence of early post-infarction remodeling, and in a secondary analysis, adverse outcome. However, it was not evaluated whether independent and incremental prognostic information was provided by the remote zone native T1 values over established CMR markers of infarct severity. Reinstadler et al. [14] reported that in addition to clinical risk factors and traditional CMR outcome markers, independent and incremental prognostic information was provided by remote zone alterations by native T1 mapping. The reproducibility of native T1 mapping is excellent with significant regional differences [15]. Thus, T1 of remote zone alterations may become a novel therapeutic target and a useful parameter for optimized risk stratification. Previous studies mainly focused on remote myocardium alterations in acute phase and do not evaluate the impact of MVO on remote myocardium alterations [14, 16]. Consequently, the promising role of dynamic remote myocardium alterations with MVO for the prediction of hard clinical events, and especially its potential incremental prognostic value compared with other markers of infarct severity, remains uncertain.

We designed a longitudinal clinical study in which patients with STEMI successfully treated by primary angioplasty were prospectively recruited, and a CMR was performed within the first week post-reperfusion, on day 30, and at 6 months. The impact of the dynamic change on post-MI CMR measures of remote myocardium was evaluated in reperfused MI by performing T1 mapping and a comprehensive serial CMR imaging study. Our study aimed to investigate the correlations between T1 mapping in remote myocardium and microvascular impairment and to comprehensively assess the value of T1 mapping as a prognostic indicator in patients with STEMI treated by primary percutaneous coronary intervention (PPCI).

## Materials and methods

### Study population and clinical endpoints

This was a prospective observational investigator-led study conducted at the Renji Hospital between June 2015 and December 2018. Patients with STEMI, who underwent PPCI within 12 h of symptom onset, were prospectively enrolled in our study.

Our study protocol was approved by the institutional ethics committee and was also in accordance with the Declaration of Helsinki. Written informed consent was obtained from all participants. Fifty age-matched normal participants were recruited as the control group to acquire the normal reference range. Exclusion criteria for participants included the commonly accepted contraindications to CMR, such as the usage of devices (implantable cardioverter-defibrillators, pacemakers, and cerebral aneurysm clips), non-ischemic cardiomyopathy (amyloidosis, cardiomyopathy due to iron deposition, valvular heart disease, evidence of inflammatory processes or Anderson–Fabry disease, and so on), coronary artery bypass grafting, previous AMI, severe claustrophobia, estimated glomerular filtration rate < 30 ml/min/1.73 m^2^, and/or significant arrhythmias. The clinical endpoint was major adverse cardiac events (MACE) composite of death, myocardial reinfarction, malignant arrhythmia, and hospitalization for heart failure within 3 years.

### CMR imaging

CMR examinations were performed within 1 week, 30 days, and 6 months after STEMI with a 3.0 T scanner (Ingenia, Philips, Best, The Netherlands). The parameters of CMR sequences (SSFP, steady-state free-precession; T2WI-STIR, T2-weighted short-tau triple inversion recovery; native T1 mapping; T2* mapping; and first pass perfusion and LGE) are described in Additional file 1: Table S1.

### Analysis of CMR images

Cardiac MR images were analyzed by two radiologists from our team with at least 5 years’ experience (Lian-Ming Wu and Bing-Hua Chen, with over 10 and 7 years) in CMR diagnostic imaging. The LV functional parameters (volumes, mass, and function) and morphology were qualified and quantified with commercial software Cvi42 Version 5.11.3 (Circle Cardiovascular Imaging Inc., Calgary, Canada). We defined remote myocardium as myocardium without evidence of infarction, edema, or enhancement [13]. The ROI of T1 mapping was placed in the territory of non-culprit vessel, which located in the myocardial segment 180°from the infarcted territory [17]. ROI in the remote myocardium was taken in the same place across the 3 scans.

Transmurality was estimated by using the centerline chord method as previous study proposed during the acute stage [18]. Microvascular obstruction (MVO) refers to a dark hypointense core within the regions of hyperenhancement on LGE during the first week after reperfusion. Intramyocardial hemorrhage (IMH) is defined as a hypointense core within the myocardial infarction zone on T2* mapping [17, 19]. Special care should be taken to have sufficient distance from adjacent tissue, such as the lungs or blood, to avoid partial volume artifacts [20]. The infarct volume fraction was quantified in LGE with full-width at half-maximum (FWHM) technique [21].

### Statistical analysis

Statistical analysis was performed using SPSS Statistics version 23.0, (IBM SPSS Inc., Chicago, Illinois, USA) and MedCalc Software version 11.4.2.0 (MedCalc Software, Ostend, Belgium). Normality of variable distribution was determined by the Kolmogorov–Smirnov test and qualitative inspection of Q–Q plots. Continuous variables with normal distribution, expressed as mean ± SD, were compared using the independent t test of two samples. Non-normally distributed variables, expressed as median and inter-quartile ranges, were compared with Kruskal–Wallis test. Frequency (percentage) for categorical data of different groups was compared using the chi-square test or Fisher exact test. The optimal cutoff values of remote myocardial T1 for the prediction of MACE were calculated by the Youden index value. MACE-free survival was described by the Kaplan–Meier method. To identify predictors of MACE, we performed univariable and multivariable Cox regression analyses. To determine independent associations with MACE during follow-up (adjusted hazard ratio [95% CI]), we performed multivariable analysis with the forward selection (likelihood ratio) modeling. Five separate models were performed to ensure statistical robustness of the Cox regression analysis. C-statistics were used to compare different parameters for predicting MACE. A 2-tailed *p* < 0.05 was considered statistically significant.

## Results

### Study population

**Fig. 1:**
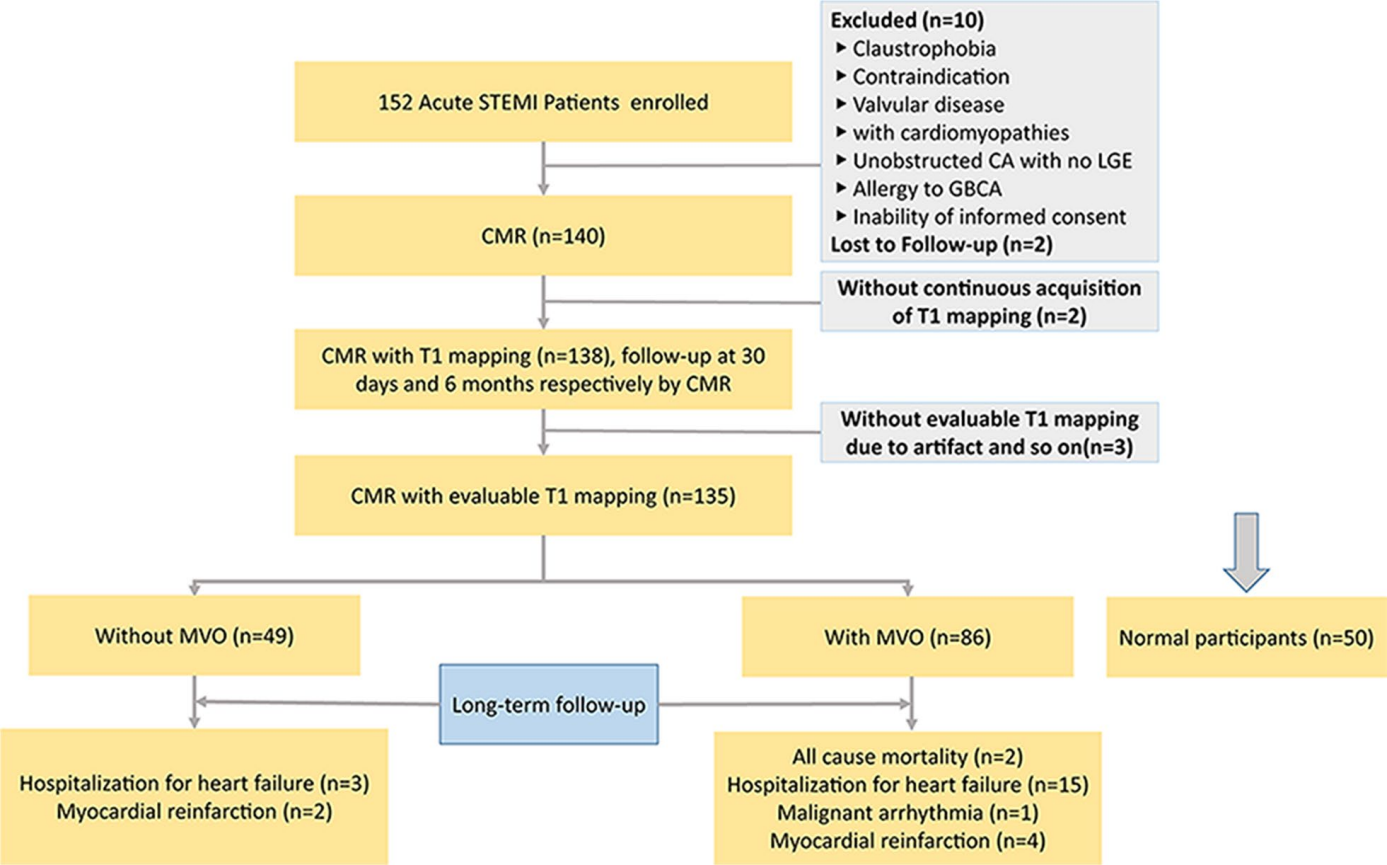
Study flowchart. A total of 135 participants were available at the end of our present analysis. Fifty normal participants were recruited as the control group

Of 152 patients with STEMI included in our prospective observational study, 135 patients (mean age 60.72 years; 12.70% female, median follow-up of 510 days) underwent the standardized CMR protocol at least twice (Fig. 1). Overall, 86 patients with MVO and 49 patients without MVO were followed up for three years. Participants were dichotomized according to whether they were complicated with MVO (86 patients with MVO).

### Baseline characteristics

The patients’ baseline characteristics are summarized in Table 1. Patients with MVO tended to have higher peaks of CK-MB (*p* = 0.006), cTnI (*p* < 0.001), BNP (*p* = 0.020), and CRP (*p* < 0.001) and were more likely to have MACE (25.60% vs. 8.20%, *p* = 0.013). The obstruction of culprit vessels occurred more often in the proximal segments in patients with MVO than without MVO (*p* = 0.002). TIMI flow grade was lower in patients with MVO during pre (*p* = 0.030)- and post-PCI (*p* = 0.040).

**Table 1:** Patients baseline characteristics

	Total (*n* = 135)	MVO(-) (*n* = 49)	MVO( +) (*n* = 86)	*p* value
Age, years	60.72 ± 10.73	62.35 ± 8.44	59.79 ± 11.78	0.150
Female	17 (12.70%)	8 (16.30%)	9 (10.50%)	0.320
Body surface area, m^2^	1.82 ± 0.16	1.79 ± 0.16	1.84 ± 0.16	0.100
Body mass index, kg/m^2^	24.51 ± 2.86	23.86 ± 2.34	24.87 ± 3.07	0.050
Chest distress	64 (47.40%)	21 (42.90%)	43 (50%)	0.420
Chest pain	126 (93.30%)	45 (91.80%)	81 (94.20%)	0.870
Risk factors				
Hypertension	71 (54.80%)	23 (46.90%)	51 (59.30%)	0.170
Diabetes mellitus (II)	47 (34.80%)	13 (26.50%)	34 (39.50%)	0.130
Killip class				0.710
I	118 (87.40%)	44 (89.80%)	74 (86%)	
II	11 (8.10%)	4 (8.20%)	7 (8.10%)	
III–IV	6 (4.40%)	1 (2%)	5 (5.80%)	
CK-MB_max_, U/L	100.45 (61.70, 245.55)	69 (39.70, 189.10)	112.90 (80, 303)	0.006
cTnI_max_, ng/mL	30 (14.38, 78.25)	14.40 (6.57, 22.80)	57 (25.50, 83)	< 0.001
BNP_max_, pg/mL	304 (135.75, 482.25)	211 (100.50,459)	350 (159.50, 496.50)	0.020
CRP_max_, mg/L	10.05 (4.67, 16.86)	5.90 (2.15, 12.65)	11.90 (7.70, 23.04)	< 0.001
MACE	26 (19.26%)	4 (8.20%)	22 (25.60%)	0.013
Angiographic				
Infarct-related artery				0.360
LAD	101 (74.80%)	35 (71.40%)	66 (76.70%)	
LCX	6 (4.40%)	1 (2%)	5 (5.80%)	
RCA	28 (20.70%)	13 (26.50%)	15 (17.40%)	
Segment of culprit vessel				0.002
Proximal	74 (54.80%)	19 (38.80%)	56 (64%)	
Middle	49 (36.30%)	21 (42.90%)	28 (32.60%)	
Distal	12 (8.90%)	9 (18.40%)	3 (3.50%)	
TIMI flow grade pre-PCI				0.030
0	89 (65.90%)	24 (49%)	65 (75.60%)	
1	10 (7.40%)	3 (6.10%)	7 (8.10%)	
2	11 (8.10%)	7 (14.30%)	4 (4.70%)	
3	25 (18.50%)	15 (30.60%)	10 (11.60%)	
TIMI flow grade post-PCI				0.040
1–2	26 (19.30%)	5 (10.20%)	21 (24.40%)	
3	10,980.70%)	44 (89.80%)	68 (75.60%)	

Numbers are given as median (inter-quartile ranges) or mean ± standard deviation or as absolute frequency with percentages in parentheses. *p* value represents comparison of patients with MVO and without MVO

cTnI_max_, peak troponin I; CK-MB_max_, peak creatinine kinase-MB; BNP_max_, peak brain natriuretic peptide; CRP_max_, peak c-creative protein; LAD, left anterior descending; RCA, right coronary artery; LCX, left circumflex; TIMI, thrombolysis in myocardial infarction, PCI, percutaneous coronary intervention; MACE, major adverse cardiovascular events; TIMI flow grade post-PCI (1–2); and TIMI flow was 1 or 2 after primary PCI

### CMR findings

Patients with MVO tended to have lower LVEF on three occasions: within 1 week (*p* = 0.001), 30 days (*p* = 0.001), and 6 months (*p* = 0.003). They were also more likely to have higher LGE volume on three occasions: within 1 week (*p* < 0.001), 30 days (*p* < 0.001), and 6 months (*p* = 0.002) (Table 2). Transmural infarction (*p* < 0.001), pericardial effusion (*p* < 0.001), and IMH (*p* < 0.001) occurred more often in patients with MVO.

**Table 2:** Left ventricular function and tissue characteristics of CMR in dynamic evolution

	Total (*n* = 135)	MVO(−) (*n* = 49)	MVO(+) (*n* = 86)	*p* value
LVEDV, mL				
1 week	130. 56 (107.89, 151.79)	116.87 (99.54, 134)	141.17 (118.62, 159.38)	< 0.001
Day 30	132.60 (111, 161.50)	123 (102.47, 147)	143 (124, 179.16)	0.001
6 Months	142.37 (113.90, 172)	127.43 (107.50, 158.76)	155.30 (117.17, 193.48)	0.020
LVESV, mL				
1 week	60 (49.10, 83.63)	51.32 (41.56, 65)	71.30 (52.56, 91.12)	< 0.001
Day 30	60 (43.23, 91.10)	44.47 (40.20, 72)	69.60 (51.76, 106.16)	< 0.001
6 Months	67 (49, 100.20)	58.3 (46.8, 78.6)	76 (58, 119)	0.001
SV, mL				
1 week	65.34 ± 16.94	63.63 ± 14.73	66.34 ± 18.10	0.340
Day 30	69.05 ± 16.71	67.8 ± 15	70.1 ± 18.1	0.510
6 Months	70.17 ± 16.64	72.56 ± 16.17	68.40 ± 16.90	0.220
LV mass, g/m^2^				
1 week	128.73 (106.57, 146)	112.55 (99.93, 133.95)	137.60 (117.30, 151.45)	< 0.001
Day 30	116.24 (100, 133.60)	114 (100, 126)	122 (104.11, 139.90)	0.080
6 Months	111 (96.58, 132)	107 (95.50, 118.18)	121 (98.29, 142.35)	0.070
iLVEDV, mL/m^2^				
1 week	71.05 (62.09, 80.59)	66.09 (56.55, 77.23)	74.92 (65.93, 82.50)	0.001
Day 30	74.33 (61.09, 90.65)	67.62 (55.75, 80.85)	77.82 (68.08, 96.41)	0.002
6 Months	78.02 (65.11, 98.85)	71.06 (63.51, 87.82)	84.15 (67.06, 107.37)	0.030
iLVESV, mL/m^2^				
1 week	32.85 (27.57, 45.61)	29.50 (26.25, 34.12)	39.18 (28.66, 48.15)	< 0.001
Day 30	32.45 (24.93, 49.58)	26.97 (21.12, 37.82)	40.24 (28.66, 60.36)	< 0.001
6 Months	37.23 (27.34, 57.66)	31.55 (26.29, 44.13)	43.31 (29.73, 62.93)	0.001
iSV, mL/m^2^				
1 week	35.59 ± 7.87	36 ± 6.85	35.34 ± 8.43	0.610
Day 30	37.97 ± 8.18	37.41 ± 7.47	38.44 ± 8.77	0.544
6 Months	39.16 ± 8.79	40.63 ± 8.76	38.07 ± 8.73	0.150
iLV mass, g/m^2^				
1 week	68.74 (59.42, 80)	63.68 (56.44, 74.85)	72.70 (63.46, 82.88)	0.004
Day 30	62.67 (57.51, 74)	60.28 (55.69, 72.10)	65.20 (58.23, 79.45)	0.140
6 Months	62.38 (54.17, 74.36)	57.94 (54.47, 66.76)	67.43 (53.51, 75.57)	0.070
LVEF, %				
1 week	49 (42.84, 59)	56.50 (52, 62.97)	44.63 (40, 52.65)	0.001
Day 30	54.25 (44, 60.08)	58.99 (54, 65)	47.07 (38.44, 55.47)	0.001
6 Months	51.90 (40, 60)	59.73 (52.08, 69)	46.13 (37.45, 57.19)	0.003
LGE, %				
1 week	28.27 (16.30, 38.50)	18.70 (9.50, 27.47)	30.45 (27.25, 35.98)	< 0.001
Day 30	20 (12, 31.38)	11.30 (6.35, 17.92)	26.68 (18.13, 31.75)	< 0.001
6 Months	22 (12, 30.25)	11.50 (8.03, 24.10)	22.95 (14.80, 28.42)	0.002
Native T1 mapping				
1 week	1254 (1241, 1272)	1243 (1225, 1258.50)	1259.50(1248.25, 1276)	< 0.001
Day 30	1235 (1224.25, 12,426.75)	1225 (1211, 1239)	1239 (1230, 1250)	< 0.001
6 Months	1273 (1254, 1296)	1269.50 (1230, 1289.25)	1277 (1258.25, 1298.25)	0.087
Native T1_Normal_	1217 (1188.50, 1236.75)^abc^	/	/	/
Transmural infarction	89 (65.90%)	17 (34.70%)	72 (83.70%)	< 0.001
Pericardial effusion	90 (66.70%)	22 (44.90%)	68 (79.10%)	< 0.001
IMH	89 (65.90%)	7 (14.30%)	82 (95.30%)	< 0.001

Numbers are given as median (inter-quartile ranges) or mean ± standard deviation or as absolute frequency with percentages in parentheses. *p* value represents comparison of patients with MVO and without MVO

MVO, microvascular obstruction; IMH, intramyocardial hemorrhage. LVEDV, left ventricular end-diastolic volume; LVESV, left ventricular end-systolic volume; SV, stroke volume; LVEF, left ventricular ejection fraction; iLVEDV, indexed left ventricular end-diastolic volume; and LGE, late gadolinium enhancement

^a^*p* < 0.001, T1_1w_ and T1_normal_

^b^*p* = 0.012, T1_30D_ and T1_normal_

^c^*p* < 0.001, T1_6months_ and T1_normal_

**Fig. 2:**
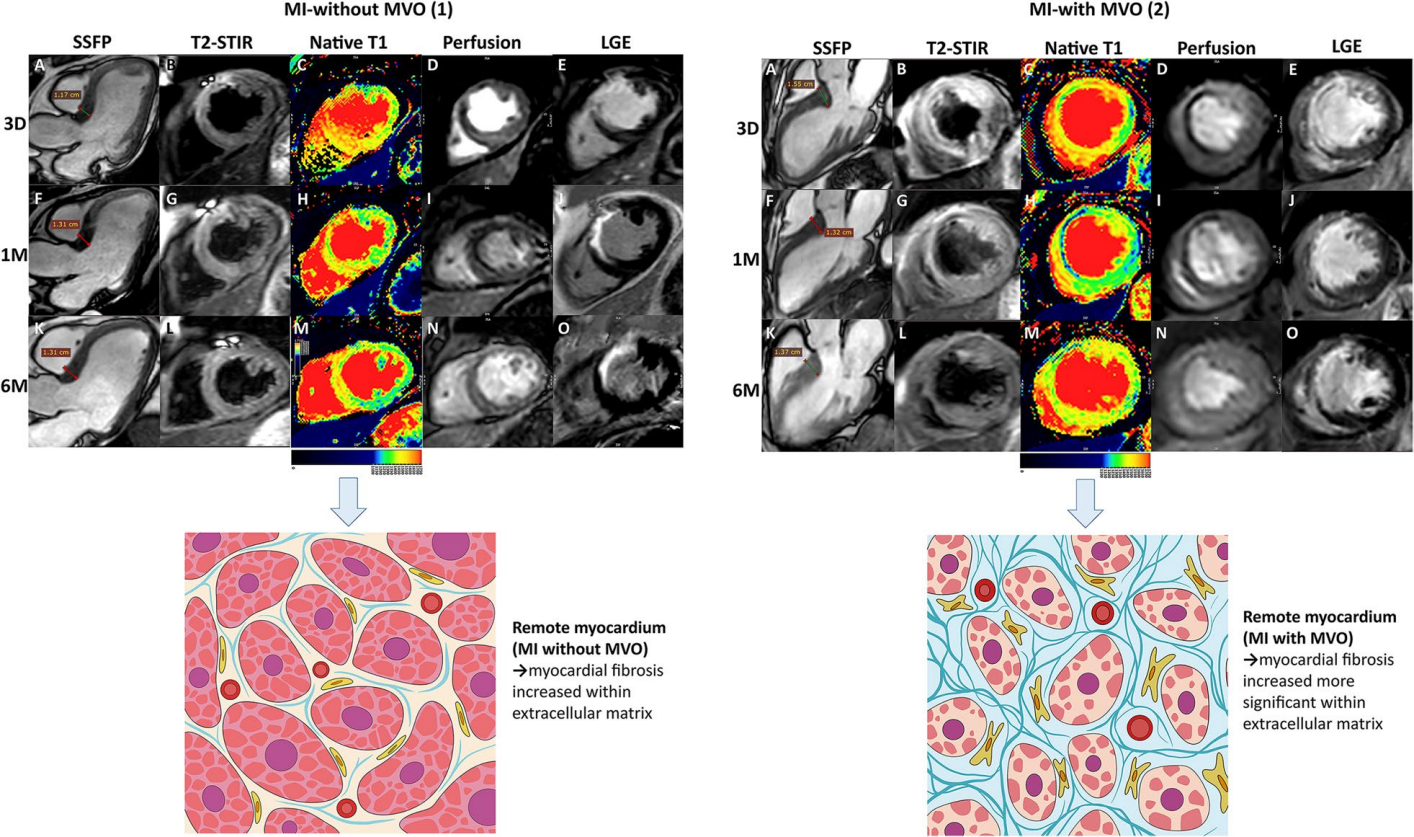
Native T1 values of remote myocardium in patients with and without MVO changed from 1 week to 6 months after MI dynamically. The native T1 value of remote myocardium in the first week was higher than those of 1 month. Inflammation of remote myocardium increased in the acute phase while diminished gradually during follow-up, and fibrosis of remote myocardium increased gradually. Native T1 values were determined by myocardial fibrosis in the chronic stage. The first patient had mural thrombosis in the apical during the first week. Compensatory thickening of the basal left ventricular septum was greater in patients with MVO than those without MVO

**Fig. 3:**
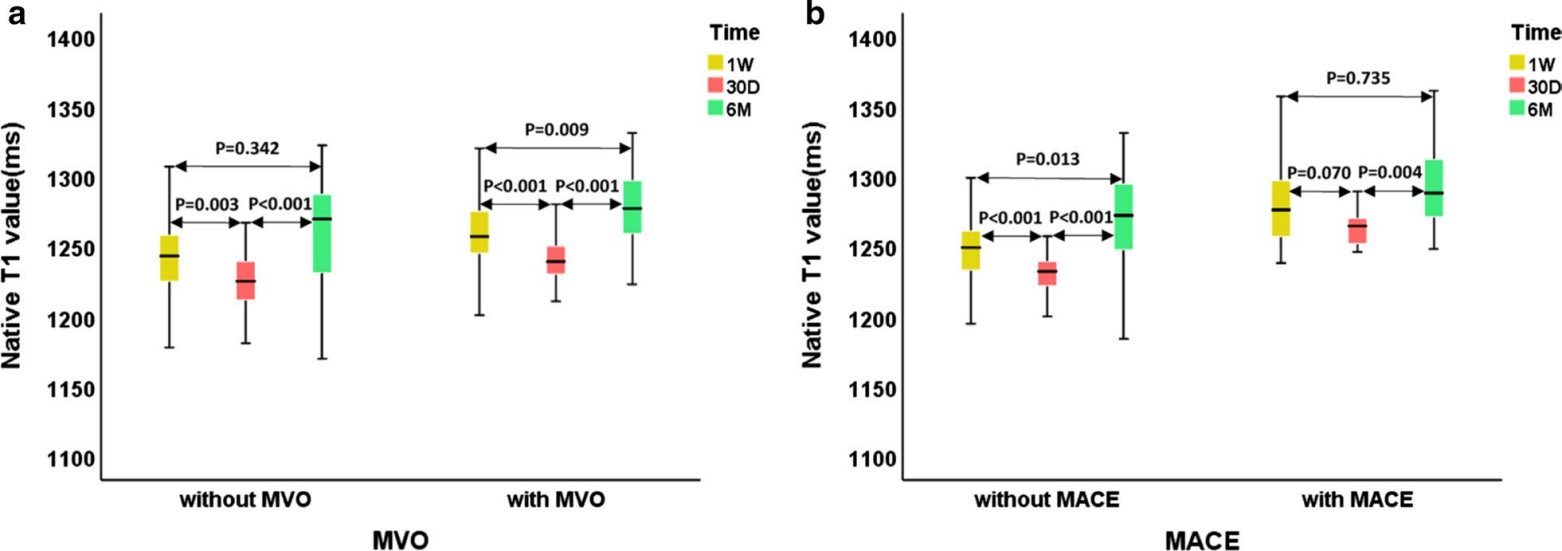
The box-plots (25th percentile, median, and 75th percentile) represent dynamic changes in native T1 values of remote myocardium on three occasions. In patients with and without MVO, remote T1 values were lowest at 30 days and highest at 6 months (**A**). In patients with and without MACE, remote T1 values were lowest at 30 days (**B**)

Native T1 values of remote myocardium in patients with and without MVO changed from 1 week to 6 months after MI dynamically. The native T1 value of remote myocardium in the first week was higher than those of 1 month. Compensatory thickening of the basal left ventricular septum was greater in patients with MVO than those without MVO (Fig. 2). At 1 week and 30 days, remote myocardium T1 values of group with MVO were higher than those without MVO (*p* = 0.030 and *p* = 0.001, respectively), while differences were not significant at 6 months (*p* = 0.09). In patients with and without MVO, remote T1 values were lowest at 30 days and highest at 6 months (Fig. 3A). In patients with and without MACE, remote T1 values were lowest at 30 days (Fig. 3B). Patients with MACE tended to have higher native T1 values (Additional file 1: Table S2).

### Endpoints and clinical outcome

During a median (510 days) follow-up, a total of 26 (19.26%) MACE (death, *n* = 2 [1.48%]; myocardial reinfarction, *n* = 4 [2.96%]; ventricular tachycardia, *n* = 1 [0.74%]; and hospitalization for heart failure, *n* = 19 [14.07%]) were observed.

In the univariable Cox regression analysis (Table 3), patients with the following characteristics were significantly associated with MACE: higher Killip class (HR 2.47, 95%CI 1.53–4, *p* < 0.001), higher cTnI_max_ (HR 1.01, 95%CI 1.001–1.02, *p* = 0.030), higher CRP_max_ (HR 1.02, 95%CI 1.01–1.03, *p* = 0.004), lower TIMI flow grade of post-PCI (HR 0.37, 95%CI 0.20–0.70, *p* = 0.002), lower LVEF_1w_ (HR 0.93, 95%CI 0.90–0.97, *p* < 0.001), lower LVEF_6M_ (HR 0.95, 95%CI 0.92–0.99, *p* = 0.013), higher LGE_1w_ (HR 1.10, 95%CI 1.06–1.14, *p* < 0.001), higher LGE_30D_ (HR 1.12, 95%CI 1.08–1.17, *p* < 0.001), higher LGE_6M_ (HR 1.05, 95%CI 1.01–1.09, *p* < 0.001), higher remote native_1w_ T1 (HR 1.02, 95%CI 1.004–1.03, *p* = 0.009), higher remote native_30D_ T1 (HR 1.04, 95%CI 1.03–1.05, *p* < 0.001), higher remote native_6M_ T1 (HR 1.02, 95%CI 1.01–1.03, *p* = 0.004), higher frequency of transmural infarction (HR 5.26, 95%CI 1.24–22.27, *p* = 0.024), IMH (HR 3.57, 95%CI 1.07–11.93, *p* = 0.038), and MVO (HR 2.95, 95%CI 1.01–8.56, *p* = 0.047).

**Table 3:** Clinical and CMR predictors of MACE in univariable cox regression analysis for patients with and without MVO

	Univariable analysis	
	Unadj HR (95% CI)	*p* value
*Clinical parameters*		
Killip class	2.47 (1.53, 4)	< 0.001
cTnI_max_, ng/mL	1.01 (1.001, 1.02)	0.030
BNP_max_, pg/mL	1.001 (1, 1.002)	0.030
CRP_max_, mg/L	1.02 (1.01, 1.03)	0.004
TIMI flow grade pre-PCI	0.90 (0.65, 1.24)	0.505
TIMI flow grade post-PCI	0.37 (0.20, 0.70)	0.002
*CMR imaging parameters*		
LVEDV_1w_, mL	1.01 (1, 1.02)	0.054
LVESV_1w_, mL	1.01 (1.002, 1.02)	0.017
iLVEDV_1w_, mL/m^2^	1.01 (0.99, 1.03)	0.211
iLVESV_1w_, mL/m^2^	1.02 (1, 1.03)	0.060
LVEF_1w_, %	0.93 (0.90, 0.97)	< 0.001
LVEF_30D_, %	0.97 (0.93, 1.01)	0.142
LVEF_6M_, %	0.95 (0.92, 0.99)	0.013
LGE_1w_, %	1.10 (1.06, 1.14)	< 0.001
LGE_30D_, %	1.12 (1.08, 1.17)	< 0.001
LGE_6M_, %	1.05 (1.01, 1.09)	< 0.001
Native_1w_ T1	1.03 (1.02, 1.04)	< 0.001
Native_30D_ T1	1.04 (1.03, 1.05)	< 0.001
Native_6M_ T1	1.02 (1.01, 1.03)	0.004
Transmural infarction	5.26 (1.24, 22.27)	0.024
Pericardial effusion	3 (0.90, 10)	0.074
IMH	3.57 (1.07, 11.93)	0.038
MVO	2.95 (1.01, 8.56)	0.047

Unadj HR, unadjusted hazard ratio; CI, confidence interval; cTnI_max_,peak troponin I; CK-MB_max_, peak creatinine kinase-MB; BNP_max_, peak brain natriuretic peptide; CRP_max_, peak c-creative protein; TIMI flow grade post-PCI (1–2); TIMI flow was 1 or 2 after primary PCI; MVO, microvascular obstruction; IMH, intramyocardial hemorrhage; LVEDV, left ventricular end-diastolic volume; LVESV, left ventricular end-systolic volume; SV, stroke volume; LVEF, left ventricular ejection fraction; iLVEDV, indexed left ventricular end-diastolic volume; and LGE, late gadolinium enhancement

**Fig. 4:**
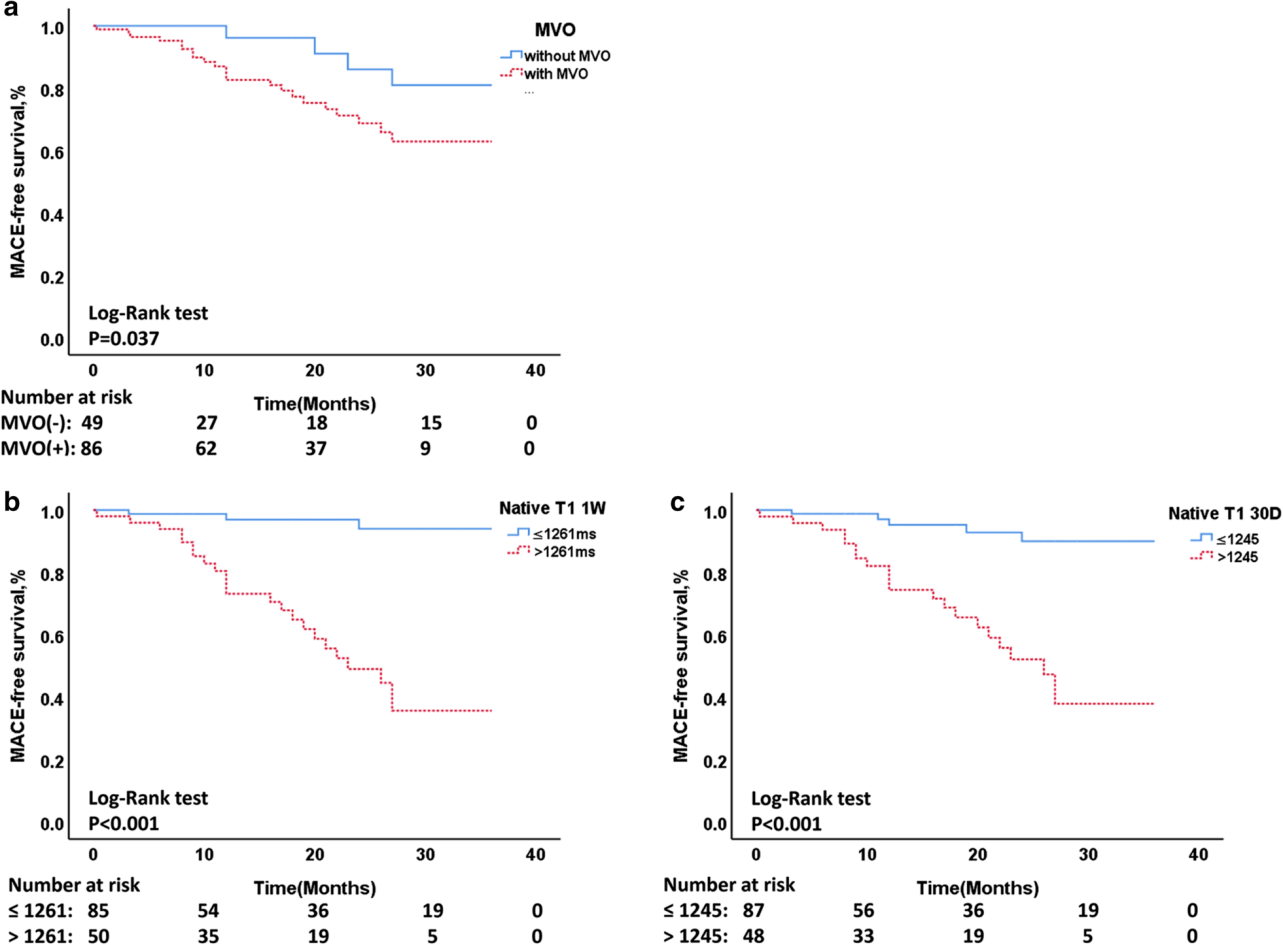
Kaplan–Meier curve for the MACE-free survival rate of three groups. **a** MVO present versus absent (*p* = 0.037). **b** Native T1_1w_ > 1261 ms versus ≤ 1261 ms (*p* < 0.001); **c** Native T1_30D_ > 1245 ms versus ≤ 1245 ms (*p* < 0.001)

For univariable Cox regression analysis, *p* ≤ 0.1 was included into stepwise multivariable Cox regression analysis. Native_1w_ T1 (HR 1.03, 95%CI 1.01–1.04, *p* = 0.002), Native_30D_ T1 (HR 1.05, 95%CI 1.03–1.07, *p* < 0.001), and LGE (HR 1.10, 95%CI 1.05–1.15, *p* < 0.001) were joint independent predictors of MACE during mid-term follow-up for all patients with STEMI after PPCI (Table 4). In multivariable cox regression analysis of 86 patients with MVO, native_30D_ T1 (HR 1.05, 95%CI 1.04–1.07, *p* < 0.001) and LGE (HR 1.10, 95%CI 1.05–1.15, *p* < 0.001) were joint independent predictors of MACE (Additional file 1: Table S4). Native T1 of remote myocardium surpasses predictive value of MVO or LGE in C-statistics of all patients (*p* < 0.001). And native_30D_ T1 value was stronger independent predictor than native_1w_ T1 in C-statistics of patients with MVO (*p* < 0.001) (Fig. 4).

**Table 4:** Clinical and CMR predictors of MACE in multivariable cox regression analysis for all patients

Model	Multivariable analysis			
	LR Chi-square (*p* value)	Variable	Adj HR (95% CI)	*p* value
Model 1	19.26 (*p* < 0.001)	Killip class	–	–
		cTnI_max_	–	–
		BNP_max_	–	–
		CRP_max_	1.01 (1, 1.03)	0.043
		TIMI flow grade post-PCI	–	–
		LVEDV	–	–
		LVESV	–	–
		iLVESV	–	–
		Transmural infarction	–	–
		Pericardial effusion	–	–
		MVO	–	–
		IMH	–	–
		LVEF_1w_	0.94 (0.90, 0.97)	< 0.001
Model 2	69.31 (*p* < 0.001)	Killip class	–	–
		cTnI_max_	–	–
		BNP_max_	–	–
		CRP_max_	–	–
		TIMI flow grade post-PCI	0.30 (0.15, 0.60)	0.001
		LVEDV	–	–
		LVESV	1.01 (1, 1.02)	0.012
		iLVESV	–	–
		Transmural infarction	–	–
		Pericardial effusion	–	–
		MVO	–	–
		IMH	–	–
		LVEF_1w_	–	–
		Native_1w_ T1	1.03 (1.02, 1.05)	< 0.001
		Native_30D_ T1	1.05 (1.03, 1.07)	< 0.001
Model 3	44.88 (*p* < 0.001)	Killip class	1.85 (1.13, 3.03)	0.015
		cTnI_max_	–	–
		BNP_max_	–	–
		CRP_max_	1.02 (1.01, 1.04)	0.004
		TIMI flow grade post-PCI	–	–
		LVEDV	–	–
		LVESV	–	–
		iLVESV	–	–
		Transmural infarction	–	–
		Pericardial effusion	–	–
		MVO	–	–
		IMH	–	–
		LGE_1w_	1.11 (1.06, 1.16)	< 0.001
Model 4	79.42 (*p* < 0.001)	Killip class	–	–
		cTnI_max_	–	–
		BNP_max_	–	–
		CRP_max_	–	–
		TIMI flow grade post-PCI	–	–
		LVEDV	–	–
		LVESV	–	–
		iLVESV	–	–
		Transmural infarction	–	–
		Pericardial effusion	–	–
		MVO	–	–
		IMH	–	–
		LGE_1w_	1.10 (1.05, 1.15)	< 0.001
		Native_1w_ T1	1.03 (1.01, 1.04)	0.002
		Native_30D_ T1	1.05 (1.03, 1.07)	< 0.001
Model 5	55.75 (*p* < 0.001)	Native_1w_ T1	1.03 (1.02, 1.05)	< 0.001
		Native_30D_ T1	1.05 (1.03, 1.06)	< 0.001
		Killip class	–	–
		BNP_max_	–	–
		TIMI flow grade post-PCI	4.28 (1.83, 10.04)	0.001
		IMH	–	–

Adj HR, adjusted hazard ratio; BNP_max_, peak brain natriuretic peptide; MVO, microvascular obstruction; IMH, intramyocardial hemorrhage; LVEF, left ventricular ejection fraction; LGE, late gadolinium enhancement; LGE_1w_ was correlated with LVEF_1w_ significantly (*r* = 0.60, *p* < 0.001); IMH was significantly correlated with cTnI (*r* = 0.62, *p* < 0.001) and MVO (*r* = 0.82, *p* < 0.001), respectively; TIMI flow grade post-PCI (1–2); and TIMI flow grade was 1 or 2 after primary PCI

## Discussion

Our comprehensive, longitudinal CMR investigation demonstrated that T1 mapping of remote myocardium changed during periods of approximately 1 week, 30 days, and 6 months. Patients with MVO following reperfused STEMI showed a substantial correlation with changes in T1 mapping of the remote myocardium. The principal findings included: (1) The severity of microvascular impairment after reperfusion is correlated with the evolution of native T1 values in remote myocardium. Patients who suffered from severe microvascular impairment display higher T1 values, which is consistent with fibrosis expansion. (2) Native_1w_ T1 value, native_30D_ T1 value, and LGE were joint independent predictors of MACE during mid-term follow-up for patients with STEMI after PPCI. (3) In reperfused STEMI patients with MVO, native_30D_ T1 and LGE were joint independent predictors of MACE.

Accurate noninvasive quantification and detection of remote myocardium alteration in reperfused STEMI patients are scientifically and clinically invaluable for their usefulness in prognosis prediction. Carrick et al. [13] found that early inflammation post-MI is associated with native T1 mapping. Several studies have confirmed that the native T1 of remote myocardium in the acute stage provided independent prognostic information for cardiac recovery and MACE [13, 14, 16]. However, none of the former studies revealed differences in native T1 values between STEMI patients with and without MVO. MVO after STEMI has a trend of dynamic change, which appears in the first week and disappears by day 30. MBF of infarcted myocardium significantly increased at 6 months compared with 1 to 3 days after STEMI [22]. Our findings unequivocally demonstrate that the dynamic changes in native T1 mapping from the acute stage to approximately 30 days, and approximately 6 months.

Local and systemic inflammatory responses were triggered due to ischemia and reperfusion in the acute stage after STEMI. Aggregation of neutrophil and platelets will further obliterate the microvascular lumen, which will then induce further microvascular impairment, ultimately leading to the production of vasoconstrictors and inflammatory mediators [23]. Acute STEMI will trigger a spectrum of alterations in the remote vessels, including upregulation of the platelet-endothelial adhesion from endothelial-associated von Willebrand factor multimers and endothelial inflammatory adhesion molecules [24]. The activation of leukocyte infiltration and proinflammatory pathways is related to the response of the remote myocardium [25]. The remodeling process post-infarction has been increasingly recognized as a cause of inflammatory response [26]. Native T1 values were determined by myocardial water content and cellularity [27]. Increased T1 of myocardium with inflammatory cell infiltration was confirmed by histopathology in patients with the features of acute rejection who underwent cardiac transplant [28]. Therefore, myocardial edema and hypercellularity, due to the inflammation during the acute phase post-STEMI, are the reasons why there has been an increase in the remote myocardial native T1 in the acute stage [13, 14]. Most patients with MVO also had IMH implicated severe microvascular impairment. Persistent crystallized iron deposition from MVO complicated with IMH is directly associated with left ventricular adverse remodeling in the chronic stage after MI, infarct resorption, and proinflammatory burden [29].

Inflammation of remote myocardium increased in the acute phase while diminished gradually during follow-up, and fibrosis of remote myocardium increased gradually. Native T1 values were determined by myocardial fibrosis in the chronic stage [30]. These may be the main reasons for the bimodal behavior of the T1 value in the remote myocardium. Diffuse myocardial fibrosis can cause the increase in native T1 [11]. In the chronic phase, prolonged T1 values correlate with the severity of myocardial interstitial fibrosis [31], which has been advocated as the sensitive marker to differentiate between fibrosis and healthy myocardium [32].

Microvascular dilation and increased blood volume may cause T1 values to increase after myocardial ischemia [33]. Another key explanation for the higher T1 values at first week, compared to values at one month, may be that myocardial blood flow of remote regions increased after MI, reflecting the hyperdynamic performance of remote myocardium [34]. Coronary microvascular injury and obstruction occur in approximately half of patients after successful primary PCI and is associated with worse outcomes compared to those without microvascular injury [3]. Severe microvascular impairment will cause a stronger systemic response, including both the infarcted and non-infarcted myocardium. Compensatory enhancement of systolic function in remote myocardium leads to compensatory cellular hypertrophy, hyperkinesis after STEMI, and cellular dysfunction [11].

Our study has several limitations. First, our sample size was small. However, a total of 341 CMR scans were performed (within 1 week = 135, 30 days = 134, 6 months = 72) on 135 patients, with each patient undergoing at least two scans. Thus, it is necessary to confirm our findings in a large-scale study. Nevertheless, our data—baseline characteristics, CMR results on infarct severity, and incidence of MACE—are comparable with other recent CMR studies [4]. In light of the fact that patients were scanned successively, we kept our CMR protocol as short as possible. Instead, both myocardium edema and hemorrhage were assessed by T2W-STIR imaging, a sequence validated and used in many published papers for these purposes. Patients with anterior STEMI, and also patients with inferolateral wall STEMI, were included in our clinical study. This may possibly induce magnetic-field non-homogeneity associated with the inferolateral wall. Additionally, care should be taken when extrapolating results to lateral MI locations, especially when attempting to adequately visualize the phenomenon, as signal loss attributable to through-plane cardiac motion may occur [35]. Wall stress may affect the pathophysiology of the remote zone in STEMI. Future studies are needed to examine wall stress and determine the clinical significance of native T1 changes in the longer-term follow-up.

To summarize, our study is the first longitudinal pathophysiological study of remote myocardium that evaluated mid-term outcomes through CMR. Native T1 mapping could detect myocardial abnormalities in remote myocardium that may be neglected by conventional LGE. The early detection of diffuse myocardial pathological alterations in the remote myocardium among survivors of STEMI allows for a more timely and individualized treatment and disease-specific therapy. The assessment of dynamic evolution of remote myocardium and the adjudication of outcome events are other strengths of the study.

## Conclusion

The evolution of native T1 in remote myocardium associated with the extent of microvascular impairment after reperfusion injury. Native_1w_ T1 value, native_30D_ T1 value, and LGE were joint independent predictors of MACE during mid-term follow-up for all patients with STEMI after PPCI. In reperfused STEMI patients with MVO, native_30D_ T1 and LGE were joint independent predictors of MACE. These findings may provide insight into the assessment of LV remodeling and prognosis after myocardial infarction.

## Supplementary Information

**Additional file 1.** The supplementary file provides a detailed description of research methods and results.

## Data Availability

The datasets used and/or analyzed during the current study are available from the corresponding author on reasonable request.

## Abbreviations

AMI: Acute myocardial infarction
BNP_max_: Peak brain natriuretic peptide
CK-MB_max_: Peak creatinine kinase-MB
CMR: Cardiac magnetic resonance
CRP_max_: Peak C-creative protein
cTnI_max_: Peak troponin I
iLVEDV: Indexed left ventricular end-diastolic volume
IMH: Intramyocardial hemorrhage
LAD: Left anterior descending
LGE: Late gadolinium enhancement
LVEDV: Left ventricular end-diastolic volume
LVEF: Left ventricular ejection fraction
LVESV: Left ventricular end-systolic volume
MACE: Major adverse cardiac events
MVO: Microvascular obstruction
NT-proBNP: N-terminal pro-brain natriuretic peptide
PPCI: Primary percutaneous coronary intervention
SSFP: Steady-state free-precession
STEMI: ST-segment elevation myocardial infarction
T2WI-STIR: T2-weighted short-tau triple inversion recovery

## References

[1] Rodriguez-Palomares JF, Gavara J, Ferreira-Gonzalez I, Valente F, Rios C, Rodriguez-Garcia J, Bonanad C, Garcia Del Blanco B, Minana G, Mutuberria M, Nunez J, Barrabes J, Evangelista A, Bodi V, Garcia-Dorado D (2019). Prognostic value of initial left ventricular remodeling in patients with reperfused STEMI. JACC Cardiovasc Imaging.

[2] Yellon DM, Hausenloy DJ (2007). Myocardial reperfusion injury. N Engl J Med.

[3] Niccoli G, Scalone G, Lerman A, Crea F (2016). Coronary microvascular obstruction in acute myocardial infarction. Eur Heart J.

[4] Eitel I, de Waha S, Wohrle J, Fuernau G, Lurz P, Pauschinger M, Desch S, Schuler G, Thiele H (2014). Comprehensive prognosis assessment by CMR imaging after ST-segment elevation myocardial infarction. J Am Coll Cardiol.

[5] de Waha S, Desch S, Eitel I, Fuernau G, Lurz P, Leuschner A, Grothoff M, Gutberlet M, Schuler G, Thiele H (2012). Relationship and prognostic value of microvascular obstruction and infarct size in ST-elevation myocardial infarction as visualized by magnetic resonance imaging. Clin Res Cardiol.

[6] Hadamitzky M, Langhans B, Hausleiter J, Sonne C, Byrne RA, Mehilli J, Kastrati A, Schomig A, Martinoff S, Ibrahim T (2014). Prognostic value of late gadolinium enhancement in cardiovascular magnetic resonance imaging after acute ST-elevation myocardial infarction in comparison with single-photon emission tomography using Tc99m-Sestamibi. Eur Heart J Cardiovasc Imaging.

[7] Robbers L, Nijveldt R, Beek AM, Teunissen PFA, Hollander MR, Biesbroek PS, Everaars H, van de Ven PM, Hofman MBM, van Royen N, van Rossum AC (2018). The influence of microvascular injury on native T1 and T2* relaxation values after acute myocardial infarction: implications for non-contrast-enhanced infarct assessment. Eur Radiol.

[8] Pfeffer MA, Braunwald E (1990). Ventricular remodeling after myocardial infarction. Exp Observ Clin Implic Circ.

[9] Cleutjens JP, Verluyten MJ, Smiths JF, Daemen MJ (1995). Collagen remodeling after myocardial infarction in the rat heart. Am J Pathol.

[10] Kramer CM, Rogers WJ, Theobald TM, Power TP, Geskin G, Reichek N (1997). Dissociation between changes in intramyocardial function and left ventricular volumes in the eight weeks after first anterior myocardial infarction. J Am Coll Cardiol.

[11] Bogaert J, Bosmans H, Maes A, Suetens P, Marchal G, Rademakers FE (2000). Remote myocardial dysfunction after acute anterior myocardial infarction: impact of left ventricular shape on regional function: a magnetic resonance myocardial tagging study. J Am Coll Cardiol.

[12] Husser O, Chaustre F, Sanchis J, Nunez J, Monmeneu JV, Lopez-Lereu MP, Bonanad C, Gomez C, Oltra R, Llacer A, Riegger GA, Chorro FJ, Bodi V (2012). Function of remote non-infarcted myocardium after STEMI: analysis with cardiovascular magnetic resonance. Int J Cardiovasc Imaging.

[13] Carrick D, Haig C, Rauhalammi S, Ahmed N, Mordi I, McEntegart M, Petrie MC, Eteiba H, Lindsay M, Watkins S, Hood S, Davie A, Mahrous A, Sattar N, Welsh P, Tzemos N, Radjenovic A, Ford I, Oldroyd KG, Berry C (2015). Pathophysiology of LV remodeling in survivors of STEMI: inflammation remote myocardium, and prognosis. JACC Cardiovasc imaging.

[14] Reinstadler SJ, Stiermaier T, Liebetrau J, Fuernau G, Eitel C, de Waha S, Desch S, Reil JC, Poss J, Metzler B, Lucke C, Gutberlet M, Schuler G, Thiele H, Eitel I (2018). Prognostic significance of remote myocardium alterations assessed by quantitative noncontrast T1 mapping in ST-segment elevation myocardial infarction. JACC Cardiovasc Imaging.

[15] Bottcher B, Lorbeer R, Stocklein S, Beller E, Lang CI, Weber MA, Meinel FG (2021). Global and regional test-retest reproducibility of native T1 and T2 mapping in cardiac magnetic resonance imaging. J Magn Reson Imaging.

[16] Puntmann VO, Carr-White G, Jabbour A, Yu CY, Gebker R, Kelle S, Rolf A, Zitzmann S, Peker E, D'Angelo T, Pathan F (2018). International TMCMROS, native T1 and ECV of noninfarcted myocardium and outcome in patients with coronary artery disease. J Am Coll Cardiol.

[17] Bulluck H, Dharmakumar R, Arai A, Berry C, Hausenloy D (2018). Cardiovascular magnetic resonance in acute ST-segment-elevation myocardial infarction: recent advances. Controversies Future Directions Circ.

[18] Amado LC, Gerber BL, Gupta SN, Rettmann DW, Szarf G, Schock R, Nasir K, Kraitchman DL, Lima JA (2004). Accurate and objective infarct sizing by contrast-enhanced magnetic resonance imaging in a canine myocardial infarction model. J Am Coll Cardiol.

[19] Kandler D, Lucke C, Grothoff M, Andres C, Lehmkuhl L, Nitzsche S, Riese F, Mende M, de Waha S, Desch S, Lurz P, Eitel I, Gutberlet M (2014). The relation between hypointense core, microvascular obstruction and intramyocardial haemorrhage in acute reperfused myocardial infarction assessed by cardiac magnetic resonance imaging. Eur Radiol.

[20] Puntmann VO, Peker E, Chandrashekhar Y, Nagel E (2016). T1 mapping in characterizing myocardial disease: a comprehensive review. Circ Res.

[21] Mikami Y, Kolman L, Joncas S, Stirrat J, Scholl D, Rajchl M, Lydell CP, Weeks SG, Howarth AG, White JA (2014). Accuracy and reproducibility of semi-automated late gadolinium enhancement quantification techniques in patients with hypertrophic cardiomyopathy. J Cardiovasc Magn Reson.

[22] Borlotti A, Jerosch-Herold M, Liu D, Viliani D, Bracco A, Alkhalil M, De Maria GL (2019). Acute microvascular impairment post-reperfused STEMI is reversible and has additional clinical predictive value: a CMR OxAMI study. JACC Cardiovasc Imaging.

[23] Bekkers SC, Yazdani SK, Virmani R, Waltenberger J (2010). Microvascular obstruction: underlying pathophysiology and clinical diagnosis. J Am Coll Cardiol.

[24] Moccetti F, Brown E, Xie A, Packwood W, Qi Y, Ruggeri Z, Shentu W, Chen J, Lopez JA, Lindner JR (2018). Myocardial infarction produces sustained proinflammatory endothelial activation in remote arteries. J Am Coll Cardiol.

[25] Lee WW, Marinelli B, van der Laan AM, Sena BF, Gorbatov R, Leuschner F, Dutta P, Iwamoto Y, Ueno T, Begieneman MP, Niessen HW, Piek JJ, Vinegoni C, Pittet MJ, Swirski FK, Tawakol A, Di Carli M, Weissleder R, Nahrendorf M (2012). PET/MRI of inflammation in myocardial infarction. J Am Coll Cardiol.

[26] Seropian IM, Toldo S, Van Tassell BW, Abbate A (2014). Anti-inflammatory strategies for ventricular remodeling following ST-segment elevation acute myocardial infarction. J Am Coll Cardiol.

[27] Mathur-De Vre R (1984). Biomedical implications of the relaxation behaviour of water related to NMR imaging. Br J Radiol.

[28] Miller CA, Naish JH, Shaw SM, Yonan N, Williams SG, Clark D, Bishop PW, Ainslie MP, Borg A, Coutts G, Parker GJ, Ray SG, Schmitt M (2014). Multiparametric cardiovascular magnetic resonance surveillance of acute cardiac allograft rejection and characterisation of transplantation-associated myocardial injury: a pilot study. J Cardiovasc Magn Reson.

[29] Kali A, Cokic I, Tang R, Dohnalkova A, Kovarik L, Yang HJ, Kumar A, Prato FS, Wood JC, Underhill D, Marban E, Dharmakumar R (2016). Persistent microvascular obstruction after myocardial infarction culminates in the confluence of ferric iron oxide crystals, proinflammatory burden, and adverse remodeling. Circ Cardiovasc Imaging..

[30] French BA, Kramer CM (2007). Mechanisms of post-infarct left ventricular remodeling. Drug Discov Today Dis Mech.

[31] Bull S, White SK, Piechnik SK, Flett AS, Ferreira VM, Loudon M, Francis JM, Karamitsos TD, Prendergast BD, Robson MD, Neubauer S, Moon JC, Myerson SG (2013). Human non-contrast T1 values and correlation with histology in diffuse fibrosis. Heart.

[32] Puntmann VO, Voigt T, Chen Z, Mayr M, Karim R, Rhode K, Pastor A, Carr-White G, Razavi R, Schaeffter T, Nagel E (2013). Native T1 mapping in differentiation of normal myocardium from diffuse disease in hypertrophic and dilated cardiomyopathy. JACC Cardiovasc Imaging.

[33] Mahmod M, Piechnik SK, Levelt E, Ferreira VM, Francis JM, Lewis A, Pal N, Dass S, Ashrafian H, Neubauer S, Karamitsos TD (2014). Adenosine stress native T1 mapping in severe aortic stenosis: evidence for a role of the intravascular compartment on myocardial T1 values. J Cardiovasc Magn Reson.

[34] Rechavia E, de Silva R, Nihoyannopoulos P, Lammertsma AA, Jones T, Maseri A (1995). Hyperdynamic performance of remote myocardium in acute infarction. Correlation between regional contractile function and myocardial perfusion. Eur Heart J.

[35] Fernandez-Friera L, Garcia-Ruiz JM, Garcia-Alvarez A, Fernandez-Jimenez R, Sanchez-Gonzalez J, Rossello X, Gomez-Talavera S, Lopez-Martin GJ, Pizarro G, Fuster V, Ibanez B (2017). Accuracy of area at risk quantification by cardiac magnetic resonance according to the myocardial infarction territory. Rev Esp Cardiol (Engl Ed).

