# Supplementary material for: Prognostic significance of non-infarcted myocardium correlated with microvascular impairment evaluated dynamically by native T1 mapping
Source: Insights Imaging. 2023 Mar 20;14:50. doi: 10.1186/s13244-022-01360-y (PMC10027971; doi:10.1186/s13244-022-01360-y)
Supplement: Supplementary file 1 — Additional file 1. The supplementary file provides a detailed description of research methods and results. [file 13244_2022_1360_MOESM1_ESM.pdf]

ELECTRONIC SUPPLEMENTARY MATERIAL

Prognostic Significance of Noninfarcted Myocardium Correlated with Microvascular Impairment Evaluated Dynamically by Native T1 Mapping

Clinical study

Supplementary Methods

Patient population

Hemodynamically stable consecutive for participants with a first STEMI (≥ 0.2 mV in at least two contiguous precordial leads, or ≥ 0.1mV in at least two contiguous limb leads), and undergoing PPCI were recruited prospectively. Patients were eligible if symptom onset was less than 12 hours before PPCI . Participants with previous CABG (coronary artery bypass grafts), previous PCI, severe cardiac valve disease, known cardiomyopathy, hemodynamic instability, and ≥12 hours after PCI were not recruited. Other exclusion criteria included all contraindications to cardiac MRI, including severe claustrophobia, defibrillators, implantable cardioverter defibrillator, implanted pacemakers, or other metallic implanted devices.

The clinical management in the acute stage was at the discretion of experienced and responsible physicians, aimed at reflecting guidelines and contemporary practice (such as high-dose clopidogrel; glycoprotein IIb/IIIa receptor inhibitors and loading aspiration catheters). Troponin I was determined pre-revascularization, and 6 months follow-up after MI.

Supplementary *CMR imaging protocol*

Supplementary Table 1 Cardiovascular Magnetic Resonance Parameters

Sequence	SSFP CINE	T2WI-STIR	T2* mapping	T1 mapping	FPP	LGE
In-plane resolution, mm	2*2*8	2*2*8	1.8*1.8*8	2*2*8	3*3*8	2*2*8
Slice thickness, mm	8	8	8	8	8	8

TR/TE, ms	3.20/1.60	2R-R/90	16/1.25+n*1.0	1.07/2.30	1.90/0.70	4/2
Bandwidth, Hz/pixel	1410	980	1430	1002	2155.20	830
Flip angle,°	45	90	20	20	20	25

SSFP (steady-state free-precession); T2WI-STIR (T2-weighted short-tau triple inversion recovery); FPP: first pass perfusion; LGE: late gadolinium enhancement.

Functional and anatomic parameters through SSFP sequence were analyzed. T2WI-STIR sequence was applied to qualify the border of remote myocardium. T2* mapping was performed to diagnose IMH. Native T1 mapping was achieved in three short axis orientations (apical, midventricular, basal). Native T1 mapping was performed with a MOLLI (motion-corrected modified look-locker inversion) recovery sequence. We applied the 5s- (3s) -3s scheme which was optimized for longer T1 times. MOLLI employs two inversion pulses. The two inversions acquired images for at least 5s, which was followed by a recovery of at least 3s -3s. Native T1 mapping was also performed for normal participants. The resting perfusion which was used to help identify MVO was performed. LGE was generally performed about 10 minutes after administrating gadolinium with Gd-DTPA (0.15 mmol/kg, diethylenetriaminepentacetate; Bayer Schering Pharma AG, Germany). LGE was performed to qualify the border of remote myocardium and existence of MVO, and also to exclude previously unrecognized myocardial infarction. The remote myocardium was nullified by optimizing the T1 inversion time.

Supplementary results

Supplementary Table 2 Differences between patients with MACE and without MACE

Native T1 mapping	Total	Without MACE (109)	With MACE (26)	p
1 week	1254(1241, 1272)	1249(1231.50,1262.50)	1279 (1271,1297)	<0.001
30days	1235(1224.25, 12426.75)	1232(1221.50,1239.50)	1264.50(1251.50,1270)	<0.001

6M	1273(1254, 1296)	1272(1245,1296)	1288(1268,1314.50)	0.045

Numbers are given as medians (inter-quartile ranges). P-values represent comparison between patients with MACE and without MACE. MACE: Major Adverse Cardiovascular Events.

Supplementary Table 3 Clinical and CMR Predictors of MACE in Univariable Cox Regression Analysis for patients **with MVO**.

	Univariable Analysis	
**Clinical parameters**	Unadj HR (95% CI)	P value
Killip class	5.78(2.34, 14.28)	<0.001
$cTnI_{max}$	1.01(0.99, 1.02)	0.375
BNP_{max}	1.001(1, 1.002)	0.116
CRP_{max}	1.02(1.002, 1.031)	0.026
TIMI flow grade pre-PCI	1.09 (0.76, 1.56)	0.630
TIMI flow grade post-PCI	2.87(1.24, 6.67)	0.014
**CMR imaging parameters**		
$LVEDV_{1w}$	1.01(1, 1.02)	0.130
$LVESV_{1w}$	1.01(1.003, 1.02)	0.015
$iLVEDV_{1w}$	1.01(0.99, 1.03)	0.201
$iLVESV_{1w}$	1.02(1.002, 1.037)	0.027
$LVEF_{1w}$	0.93(0.89, 0.97)	<0.001
$LVEF_{30D}$	0.98(0.94, 1.03)	0.466
$LVEF_{6M}$	0.96(0.92, 1.003)	0.068
LGE_{1w}	1.08(1.04, 1.13)	<0.001
LGE_{30D}	1.10(1.06, 1.15)	<0.001
LGE_{6M}	1.04(1.004, 1.08)	0.030
$Native_{1w}$ T1	1.02(1, 1.03)	0.007
$Native_{30D}$ T1	1.04(1.03, 1.05)	<0.001
$Native_{6M}$ T1	1.01(1, 1.03)	0.05
Transmural infarction	26.76(0.20, 3534)	0.187
Pericardial effusion	1.37(0.41, 4.65)	0.610
IMH	0.74(0.10, 5.50)	0.767

Unadj HR: Unadjusted Hazard Ratio; CI: confidence interval; $cTnI_{max}$: peak troponin I; $CK\text{-}MB_{max}$: peak creatinine kinase-MB; BNP_{max}: peak brain natriuretic peptide; CRP_{max}: peak c-creative protein; TIMI flow grade post-PCI(1-2): TIMI flow was 1 or 2 after primary PCI; MVO: microvascular obstruction; IMH: intramyocardial hemorrhage; LVEDV: left ventricular end diastolic volume; LVESV: left ventricular end systolic volume; SV: stroke volume; LVEF: left ventricular ejection fraction; iLVEDV: indexed left ventricular end diastolic volume; LGE: late gadolinium enhancement.

Supplementary Table 4 Clinical and CMR Predictors of MACE in Multivariable Cox Regression Analysis for patients **with MVO**.

	Multivariable Analysis			
Model	LR Chi-Square (p Value)	Variable	Adj HR (95% CI)	p Value
Model 1	17.81(P<0.001)	CRP_{max}		
		Killip class		
		TIMI flow grade post-PCI		
		$LVESV_{1w}$		
		$iLVESV_{1w}$		
		iSV_{1w}		
		$LVEF_{1w}$	0.92(0.88,0.96)	<0.001
Model 2	27.92(P<0.001)	CRP_{max}	1.02(1.01,1.04)	0.011
		Killip class	1.96(1.16,3.32)	0.012
		TIMI flow grade post-PCI		
		$LVESV_{1w}$		
		$iLVESV_{1w}$		
		LGE_{1w}	1.09(1.04,1.14)	<0.001
Model 3	4953(P<0.001)	CRP_{max}		
		Killip class		
		TIMI flow grade post-PCI		
		$LVESV_{1w}$	-	-
		$iLVESV_{1w}$		
		iSV_{1w}		
		LGE_{1w}	1.10(1.05,1.15)	P<0.001
		$Native_{1w}$ T1	-	-
		$Native_{30D}$ T1	1.05(1.04,1.07)	P<0.001
Model 4	44.35(P<0.001)	CRP_{max}	-	-
		Killip class	-	-
		TIMI flow grade post-PCI	-	-
		$LVESV_{1w}$	-	-
		$iLVESV_{1w}$	-	-
		iSV_{1w}	-	-
		$LVEF_{1w}$	0.92(0.88,0.9	P<0.001

		6)	
	$Native_{1w}$ T1	-	-
	$Native_{30D}$ T1	1.04(1.03,1.06)	P<0.001

Adj HR: adjusted Hazard Ratio; BNP_{max}: peak brain natriuretic peptide; MVO: microvascular obstruction; IMH: intramyocardial hemorrhage; LVEF: left ventricular ejection fraction; LGE: late gadolinium enhancement; LGE_{1w} was correlated with $LVEF_{1w}$ significantly (r=0.60, P<0.001); TIMI flow grade post-PCI (1-2): TIMI flow grade was 1 or 2 after primary PCI.

Supplementary Figure

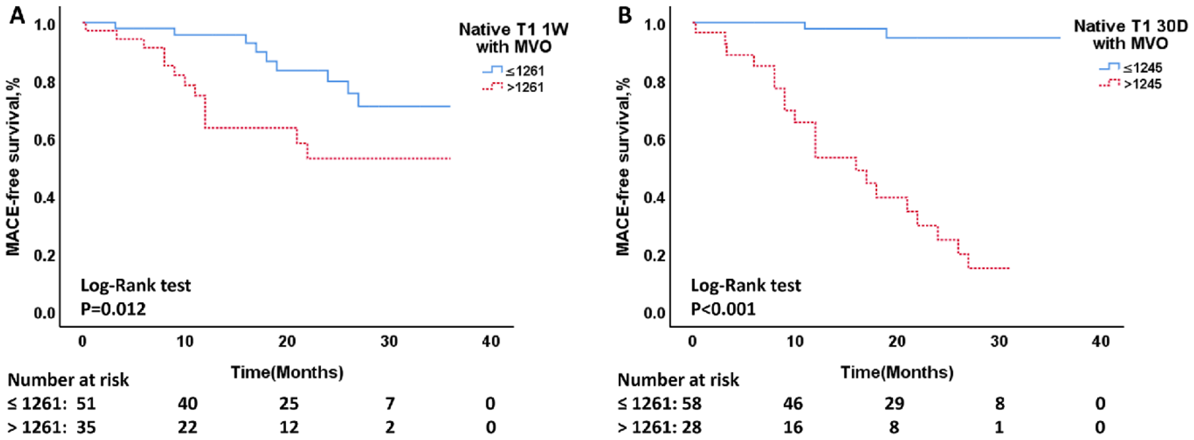

Supplementary figure 1. Kaplan-Meier curve for the MACE-free survival rate in patients with MVO after STEMI. (A). Native $T1_{1w}$ >1261 versus ≤1261 (P=0.012); (B). Native $T1_{30D}$ >1245 versus ≤1245 (P<0.001).
